# Bioaugmented Phytoremediation of Heavy Metals in Petrochemical Wastewater Using *Eichhornia crassipes*

**DOI:** 10.3390/toxics14060493

**Published:** 2026-06-05

**Authors:** Xudong Lan, Rabiya Sheraz, Waqar-Un-Nisa, Songhao Zhang, Jia Ouyang, Aansa Rukya Saleem, Jawaria Abid, Habib Ullah, Yilina Bai, Rui Ma, Shaohong You, Abubakr M. Idris, Guo Yu

**Affiliations:** 1 Guangxi Key Laboratory of Green Preparation and Application of Inorganic Materials, Guangxi Science & Technology Normal University, Laibin 546199, China; 2 Center for Interdisciplinary Research in Basic Science, International Islamic University Islamabad, Islamabad 44000, Pakistan; 3 Guangxi Key Laboratory of Theory and Technology for Environmental Pollution Control, Guilin University of Technology, Guilin 541006, China; 4 Department of Earth and Environmental Sciences, School of Engineering and Applied Sciences, Bahria University, Islamabad 44000, Pakistan; 5 Isotope Application Division, Pakistan Institute of Nuclear Science and Technology, PINSTECH, Islamabad 44000, Pakistan; 6 School of Intelligent Electrical Engineering and New Energy, Hengxing University of Science and Technology, Qingdao 266042, China; habib901@zju.edu.cn; 7 Chongqing Academy of Agricultural Sciences, Chongqing 401329, China; 8 Department of Chemistry, College of Science, King Khalid University, Abha 62529, Saudi Arabia; dramidris@gmail.com; 9 Research Center for Advanced Materials Science (RCAMS), King Khalid University, Abha 62529, Saudi Arabia; 10 Center for Water and Ecology, State Key Laboratory of Iron and Steel Industry Environmental Protection, School of Environment, Tsinghua University, Beijing 100084, China

**Keywords:** produced wastewater, phytoremediation, heavy metals, *Eichhornia crassipes*, petroleum-degrading microbes

## Abstract

This study investigated the potential of microbial-assisted phytoremediation using *Eichhornia crassipes* (water hyacinth) to reduce heavy metal and salinity pollution in produced water collected from Aadi Oil Field in Gujar Khan, Pakistan. Produced water was analyzed for physicochemical parameters and heavy metal content using Inductively Coupled Plasma–Optical Emission Spectrometry (ICP-OES) to establish baseline data. *E. crassipes* plants augmented with indigenous, contaminant-tolerant microbial isolates were employed in a 15-day laboratory experiment. The results showed a resilient growth response, with plant height increasing to approximately 11–15 cm and root length extending up to 10–13 cm across treatments. Biomass also improved, with wet weights reaching 21–24 g from an initial 20 g. The treatment effectively reduced key physicochemical parameters: pH was stabilized from an initial alkaline value of 9.14 to near-neutral values (7.0–7.5), and total dissolved solids (TDSs) were reduced by approximately 50%. Heavy metal removal rates varied, with the highest efficiency of 79.2% for Silver (Ag) and the lowest (18.5%) for Mercury (Hg) This study demonstrates that *E. crassipes* actively participated in phytoremediation by absorbing and accumulating heavy metals and reducing salinity. The association with contaminant-tolerant microbes appeared to enhance the plant’s tolerance and overall treatment efficacy, indicating that plant–microbe interactions offer a sustainable strategy for the treatment of produced water.

## 1. Introduction

Water quality is a major driver of aquatic species community composition and ecosystem service function and is vital to ensure that water is safe for drinking and recreational activities worldwide [1]. The global energy demand has led to a significant increase in oil and gas exploration and production activities, which inevitably generate massive volumes of wastewater. This byproduct, commonly referred to as produced water or produced wastewater, represents the largest volume of waste associated with petroleum extraction [2,3]. Produced water is a complex and hazardous effluent brought to the surface along with hydrocarbons, and its chemical composition is highly variable, depending on the geological location, reservoir depth, and extraction techniques [2]. Globally, the industry generates an estimated 250 million barrels of produced water daily, vastly exceeding the volume of oil produced [4]. The discharge of this wastewater, which is characterized by high concentrations of hydrocarbons, heavy metals (such as Fe, Pb, Cd, Cr, and Ni), elevated salinity (salts), and persistent organic pollutants (POPs), poses a severe threat to aquatic ecosystems, soil quality, and human health [2,5]).

The challenge is particularly acute in regions like Gujar Khan, Pakistan, where Aadi Oil Field operates. The improper disposal of produced water has led to environmental degradation, specifically the accumulation of heavy metals and high salinity in local water bodies, creating an urgent need for effective and localized remediation strategies.

Conventional methods for treating produced water, such as chemical precipitation, coagulation–flocculation, and membrane filtration (e.g., reverse osmosis), are often effective but suffer from significant drawbacks. These processes are typically energy-intensive and costly to implement and maintain and frequently result in the generation of secondary waste streams, such as toxic sludge, which requires further specialized disposal. The limitations of these traditional approaches have spurred research into more environmentally friendly, cost-effective, and sustainable alternatives. Such strategies support environmental sustainability by enabling the reuse of organic or industrial residues for effective contaminant removal in wastewater systems [6].

Given that contaminants such as heavy metals in soils can pose severe food safety and environmental risks [7], phytoremediation—a green technology that utilizes plants to remove, degrade, or stabilize environmental contaminants—has emerged as a promising solution [8]. Among the various aquatic macrophytes, *Eichhornia crassipes* (water hyacinth) is recognized globally for its exceptional potential in wastewater treatment [9]. Its suitability stems from its rapid growth rate, high biomass production, and an extensive, fibrous root system that provides a large surface area for the absorption and accumulation of pollutants, including heavy metals and excess nutrients [10]. The plant’s ability to thrive in nutrient-rich and even moderately contaminated environments makes it an ideal candidate for treating complex industrial effluents like produced water.

However, the high toxicity and salinity of raw produced water can often inhibit plant growth and reduce the overall efficiency of phytoremediation [11]. To overcome this limitation, the concept of microbial-assisted phytoremediation has gained attraction. This integrated approach leverages the synergistic relationship between the plant and contaminant-tolerant microorganisms residing in its rhizosphere. These microbes enhance the plant’ tolerance and remediation efficacy by performing crucial functions. They can directly bind to heavy metals through biosorption and precipitation to reduce their bioavailability and toxicity [12]. Additionally, they break down complex organic pollutants like hydrocarbons into less harmful compounds through redox reactions and degradation [13]. They also provide essential nutrients to the plant through nutrient mobilization, thereby promoting healthier growth under stress conditions [14].

Therefore, this study was designed to investigate the potential of this synergistic approach—the combination of *Eichhornia crassipes* with indigenous, contaminant-tolerant microbial isolates—for the remediation of produced water specifically sourced from the Aadi Oil Field in Gujar Khan, Pakistan. The specific objectives of this research study were to evaluate the baseline physicochemical properties and heavy metal load of produced water, isolate and characterize indigenous bacteria from the contaminated site capable of surviving in the toxic environment, and assess the effectiveness of microbial-assisted phytoremediation in reducing key parameters, including heavy metals and salinity-related indicators (TDS, EC, chlorides, and bicarbonates), over a 15-day period.

## 2. Materials and Methods

### 2.1. Water Sampling and Quality Analysis

A produced water sampling event was conducted at Aadi Oil Field in Gujar Khan, Pakistan. Five composite water samples were collected from different discharge points associated with oil extraction and processing activities. Each sample was collected in triplicate using sterile, pre-cleaned polyethylene bottles with a capacity of 1 L. Samples were immediately stored in ice boxes at 4 °C and transported to the laboratory for subsequent physicochemical and biological analyses. This sampling strategy ensured representative coverage of site variability and provided reliable data for evaluating the contamination levels in produced wastewater.

### 2.2. Physicochemical Parameters

The physicochemical analysis of wastewater was performed in accordance with standard methods prescribed by the American Public Health Association [10]. The parameters measured included pH, total dissolved solids (TDS), electrical conductivity (EC), bicarbonates, chlorides, sulphates, chemical oxygen demand (COD), and heavy metals. A brief description of each analytical method is provided below to ensure reproducibility.

**pH**: Recorded using a calibrated digital pH meter (Systronics, Model 335, Singapore). The meter was calibrated daily using standard buffer solutions (pH 4, 7, and 10) prior to sample analysis. Wastewater samples were equilibrated to room temperature (25 ± 2 °C) before measurement, and pH was recorded in triplicate.

**TDS:** Measured using a digital TDS meter (Systronics, Model 308, Singapore). The meter was calibrated with a standard potassium chloride solution (1000 mg/L). Samples were filtered through Whatman No. 42 filter paper to remove suspended solids, and TDSs were measured directly in the filtrate. Results are expressed in mg/L.

**EC:** Determined using a multiparameter (Eutech PCSTestr 35, Thermofisher Scientific, Waltham, MA, USA). The cell constant was verified using a standard potassium chloride solution (0.01 M, EC = 1413 µS/cm at 25 °C).

**Bicarbonates (HCO_3_^−^)**: Quantified by titration with 0.1 N hydrochloric acid using methyl orange as an indicator. The endpoint was marked by a distinct color change from yellow to orange-pink.

**Chloride (Cl^−^):** Determined by the Mohr argentometric method. A 25 mL sample was titrated against standardized 0.01 N silver nitrate (AgNO_3_) solution using 5% potassium chromate (K_2_CrO_4_) as an indicator. The endpoint was identified by the formation of a reddish-brown silver chromate precipitate.

**Sulphates (SO_4_^2−^):** Analyzed by the turbidimetric barium chloride method using a UV–visible spectrophotometer. Sulphate was precipitated as barium sulphate (BaSO_4_) by adding barium chloride crystals (BaCl_2_·2H_2_O) to an acidified sample (containing glycerol–ethanol conditioner to stabilize the suspension). Absorbance of the suspended BaSO_4_ was measured at 420 nm after 10 min of reaction time. Concentration was determined from a calibration curve prepared using sodium sulphate standards (0–40 mg/L SO_4_^2−^).

**COD:** Measured using the closed reflux colorimetric method involving potassium dichromate oxidation, according to APHA Standard Method 5220 D. A sample (2.5 mL) was digested with 1.5 mL of 0.01667 M potassium dichromate (K_2_Cr_2_O_7_) and 3.5 mL of sulphuric acid reagent (containing silver sulphate catalyst and mercuric sulphate chloride suppressor) at 150 °C for 2 h in sealed digestion tubes. After cooling, absorbance was measured at 600 nm, and COD was calculated from a potassium hydrogen phthalate (KHP) standard curve (0–1000 mg/L). Results are expressed as mg O_2_/L.

**Heavy Metals (Hg, Fe, Pb, As, Co, Cr, and Ni):** For heavy metal analysis, samples were prepared through the acid digestion method. After digestion, samples were filtered through Whatman filter paper No. 41, the filtrate was shifted into vials and diluted up to 10 mL using distilled water. The heavy metals (Hg, Fe, Pb, As, Co, Cr, and Ni) in digested samples were detected using Inductively Coupled Plasma–Optical Emission Spectrometry (ICP-OES; Agilent 5100, Santa Clara, CA, USA). The intensity of the rays within ICP-OES was used to calculate the concentration of heavy metals from the samples. Software version 7.3.1.9507 was used to run the samples on ICP-OES; the read time was 5 s, the stabilization time was 15 s, the nebulizer flow was 0.7 L/min, and the plasma flow was 12 L/min.

**Quality Assurance:** Both internal standard analysis and standard reference procedures were used to countercheck the results, which demonstrated very satisfactory recovery. The concentration of selected heavy metals was calculated in mg/L and then converted into percentage removal.

### 2.3. Bacterial Isolation and Characterization

The bacteria were isolated from the contaminated soil of the oil field from where produced water samples were collected. The soil type is silty and originated from alluvial deposits. A 200 gm composite soil sample was collected from a depth of 10–15 cm below the surface in three replicates. Soil samples were serially diluted in sterile distilled water. Aliquots from dilutions 10^−6^ to 10^−9^ were inoculated on nutrient agar plates using the spread plate technique [15] and incubated at 37 °C for 24 h. Distinct colonies were subcultured to obtain pure isolates, which were then stored at 4 °C for further studies. Bacterial characterization involved a series of biochemical tests, including Gram staining, urease activity, coagulase reaction, oxidase activity, indole production, and catalase reaction, which were performed following the protocols [16]. Gram staining helped differentiate bacterial isolates into Gram-positive or Gram-negative groups. The urease test was performed using urea broth with phenol red as an indicator, and a color change after 24 h indicated urease activity. The coagulase test involved mixing bacterial suspensions with human plasma to check for clotting. Oxidase activity was detected using Kovács reagent, Merck (Rahway, NJ, USA), while indole production was confirmed after incubation in tryptone broth followed by the addition of Kovács reagent. Catalase activity was tested by adding hydrogen peroxide to bacterial smears and observing bubble formation [17].

### 2.4. Phytoremediation Preparation

A total of 18 healthy *Eichhornia crassipes* plants were collected from a freshwater body near Islamabad, Pakistan, and initially rinsed with tap water to remove debris, algae, and attached organisms. The plants were then acclimatized for 15 days in a 1:1 mixture of produced water and clean deionized water under natural sunlight (12 h photoperiod, 25 ± 3 °C) to adapt to the chemically complex wastewater matrix prior to experimentation. This acclimatization duration aligns with established protocols in the phytoremediation literature, where 10–15-day acclimation periods are standard for *E. crassipes* to minimize transplant shock, allow for root system establishment, and activate metal tolerance mechanisms before full-strength pollutant exposure [18,19]. Following acclimatization, uniform plants (initial wet weight of 20 ± 1 g, shoot height of 8–10 cm, and root length of 6–8 cm) were randomly distributed into six treatment units (three replicates per treatment: control, plant-only, and bioaugmented plant) for the 15-day phytoremediation experiment.

### 2.5. Experimental Design

Phytoremediation experiments were conducted in 15 L plastic containers maintained at ambient temperatures between 25 and 30 °C. Six treatment setups were established, each in triplicate to ensure statistical reliability. Treatment T1 consisted of 100% produced wastewater with *E. crassipes*; T2 involved 100% wastewater, plants, and bacterial inoculum (a mixed bacterial consortium applied as a single combined inoculum, rather than individual strains); T3 included 75% wastewater with plants only (to evaluate dilution effects and plant-only remediation as a control); T4 included 75% wastewater with plants and bacteria (the same consortium inoculum); T5 combined 50% wastewater with plants; T6 contained 50% wastewater with plant and bacterial inoculum (the same consortium inoculum). The bacterial inoculum was prepared from overnight cultures adjusted to standard optical density, and 10 mL was added to each relevant container. Water samples from each treatment were collected on Day 0 and Day 15 [18]. After filtration through Whatman No. 1 filter paper, the same set of physicochemical parameters was analyzed post-treatment to assess the effectiveness of microbial-assisted phytoremediation. Upon completion of the experiment, the *E. crassipes* plants were harvested and divided into roots and shoots. Plant growth parameters, including shoot height, root length, fresh weight, and dry weight (measured after oven-drying at 100 °C to constant weight), were recorded. These data were used to evaluate the plant’s biomass production and pollutant uptake potential during the treatment period.

## 3. Results and Discussion

**Figure 1:**
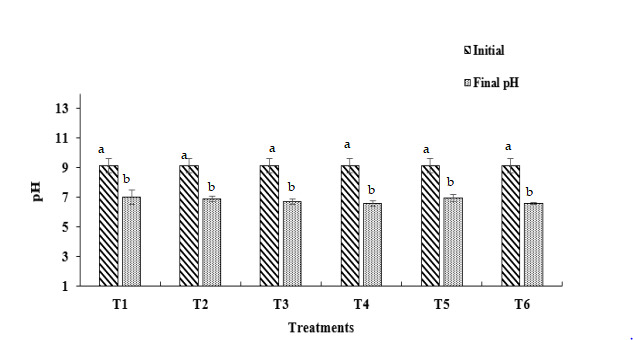
Effect of microbial-assisted phytoremediation on pH of produced wastewater. T1 = 100% produced wastewater with *E. crassipes*; T2 = 100% wastewater, plants, and bacterial inoculum; T3 = 75% wastewater with plants only (to evaluate dilution effects and plant-only remediation as a control); T4 = 75% wastewater with plants and bacteria; T5 = 50% wastewater with plants; T6 = 50% wastewater with plant and bacterial inoculum. Different lowercase letters above the bars indicate statistically significant differences among means; bars sharing the same letter are not significantly different at *p* < 0.05.

The effectiveness of microbial-assisted phytoremediation using *Eichhornia crassipes* was evaluated through physicochemical, biological, and morphological assessments. A significant reduction in pH was observed, with pH decreasing from 9.14 in untreated produced water to near-neutral values in all respective treatments. Treatment T2 (100% wastewater with plants and bacteria) demonstrated significant remediation efficiency (*p* < 0.05), with the pH decreasing by 22.8% and shifting toward neutral (Figure 1). This pH decrease may be associated with plant root exudation, microbial metabolic byproducts, CO_2_ dissolution, and buffering reactions occurring in the produced water matrix [20]. However, in the absence of abiotic controls, these explanations remain tentative and warrant further investigation. This shift is considered the most significant, as it inhibits both plant metabolism and reduces heavy metal solubility, severely limiting their bioavailability. This shift suggests that the enhanced microbial activity was facilitated by a moderate pH, which encourages the microbial degradation of contaminants [21]. The likely mechanism involves the release of organic acids by plant roots, which alters the water chemistry and supports microbial symbiosis, improving treatment outcomes [22].

**Figure 2:**
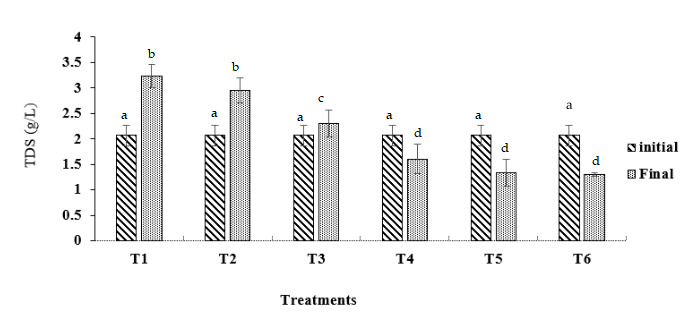
Effect of microbial-assisted phytoremediation on TDSs of produced wastewater. T1 = 100% produced wastewater with *E. crassipes*; T2 = 100% wastewater, plants, and bacterial inoculum; T3 = 75% wastewater with plants only (to evaluate dilution effects and plant-only remediation as a control); T4 = 75% wastewater with plants and bacteria; T5 = 50% wastewater with plants; T6 = 50% wastewater with plant and bacterial inoculum. Different lowercase letters above the bars indicate statistically significant differences among means; bars sharing the same letter are not significantly different at *p* < 0.05.

Total dissolved solids (TDSs) were also significantly (*p* < 0.05) reduced after treatment, reflecting the uptake of calcium and magnesium ions by plants (Figure 2). This reduction demonstrates not only the ionic absorption capability of *E. crassipes* but also a measurable improvement (approximately 50% reduction in TDS levels) in water quality, as elevated TDS levels are often linked with toxicity. These results align with findings from previous studies where aquatic macrophytes were employed to reduce dissolved solids in wastewater through nutrient and ion absorption [23].

**Figure 3:**
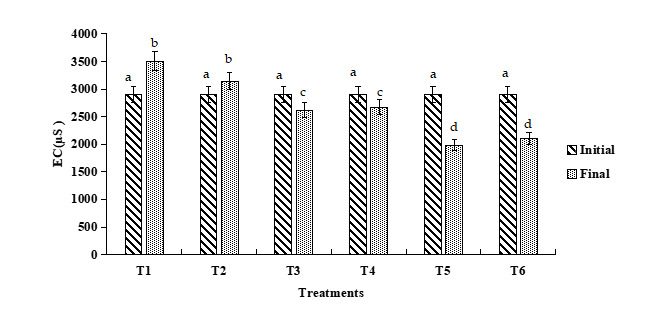
Effect of microbial-assisted phytoremediation on EC of produced wastewater. T1 = 100% produced wastewater with *E. crassipes*; T2 = 100% wastewater, plants, and bacterial inoculum; T3 = 75% wastewater with plants only (to evaluate dilution effects and plant-only remediation as a control); T4 = 75% wastewater with plants and bacteria; T5 = 50% wastewater with plants; T6 = 50% wastewater with plant and bacterial inoculum. Different lowercase letters above the bars indicate statistically significant differences among means; bars sharing the same letter are not significantly different at *p* < 0.05.

Electrical conductivity (EC), a proxy for ion concentration and salinity, showed a marked decrease following remediation. T5 and T6 exhibited the most pronounced (*p* < 0.05) EC reduction, demonstrating superior remediation efficiency at lower wastewater concentrations. This trend parallels TDS reduction, reinforcing the inference that plants and microbes removed substantial quantities of dissolved ions from water (Figure 3). These observations are consistent with Singh and Kumari’s [24], who highlighted that decreased EC values indicate improved water quality and reduced potential ecological stress on aquatic systems.

**Figure 4:**
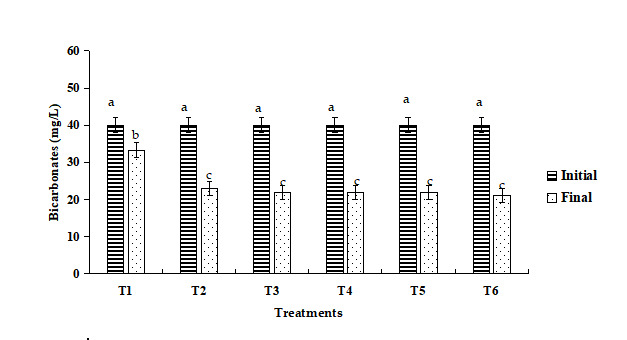
Effect of microbial-assisted phytoremediation on bicarbonates in produced wastewater. T1 = 100% produced wastewater with *E. crassipes*; T2 = 100% wastewater, plants, and bacterial inoculum; T3 = 75% wastewater with plants only (to evaluate dilution effects and plant-only remediation as a control); T4 = 75% wastewater with plants and bacteria; T5 = 50% wastewater with plants; T6 = 50% wastewater with plant and bacterial inoculum. Different lowercase letters above the bars indicate statistically significant differences among means; bars sharing the same letter are not significantly different at *p* < 0.05.

The concentration of bicarbonates declined from 32 to 15 mg/L post-treatment (Figure 4), demonstrating the plant’s role in modifying water alkalinity. This significant removal of bicarbonate ions (*p* < 0.05) likely contributed to the stabilization of pH, indicating an effective interaction between plant uptake and microbial activity. Similarly, chloride concentration was reduced from 443 mg/L to 240 mg/L, emphasizing the efficacy of *E. crassipes* in mitigating salinity.

**Figure 5:**
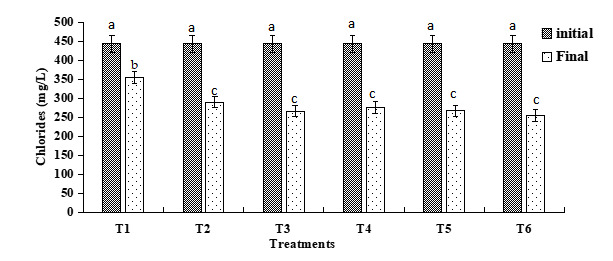
Effect of microbial-assisted phytoremediation on chloride ions of produced wastewater. T1 = 100% produced wastewater with *E. crassipes*; T2 = 100% wastewater, plants, and bacterial inoculum; T3 = 75% wastewater with plants only (to evaluate dilution effects and plant-only remediation as a control); T4 = 75% wastewater with plants and bacteria; T5 = 50% wastewater with plants; T6 = 50% wastewater with plant and bacterial inoculum. Different lowercase letters above the bars indicate statistically significant differences among means; bars sharing the same letter are not significantly different at *p* < 0.05.

All samples initially contained 443 mg/L of chloride. At the start of the experiment, the chloride level was 443 mg/L, and after the completion of the experiment, it was reduced to 240 mg/L (Figure 5). Chloride concentration decreases because plant roots absorb chloride, reducing its concentration. This significant reduction (*p* < 0.05) highlights the chloride-removal potential of *E. crassipes* during phytoremediation. Lower chloride levels contribute to decreased salinity and improved water quality, which are essential to protecting aquatic life and ensuring the suitability of water for reuse or discharge into natural ecosystems [25].

**Figure 6:**
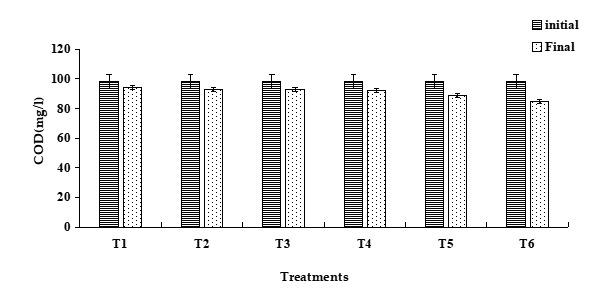
Effect of microbial-assisted phytoremediation on chemical oxygen demand of produced wastewater. T1 = 100% produced wastewater with *E. crassipes*; T2 = 100% wastewater, plants, and bacterial inoculum; T3 = 75% wastewater with plants only (to evaluate dilution effects and plant-only remediation as a control); T4 = 75% wastewater with plants and bacteria; T5 = 50% wastewater with plants; T6 = 50% wastewater with plant and bacterial inoculum.

A 20% reduction in chemical oxygen demand (COD) indicates the partial but meaningful degradation of organic matter in wastewater (Figure 6). Though lower COD values were reported in some studies [26,27], this result confirms that even moderate remediation with water hyacinth, assisted by microbes, can promote the oxygenation and breakdown of pollutants. The photosynthetic oxygen released by plants may enhance aerobic microbial activity, which facilitates the degradation of hydrocarbons and organic pollutants [28].

### 3.1. Characterization of Indigenous Oil Field Bacterial Isolates

Four indigenous bacterial strains were isolated from petroleum-contaminated soil at the oil field site and characterized using various biochemical tests (Table 1). All strains tested positive for catalase and coagulase activities but were urease-negative. The isolates were identified as Gram-positive bacteria, exhibiting distinct morphological characteristics like spiral, rod, spherical, and comma shapes. These bacteria are likely involved in the breakdown of contaminants in wastewater.

### 3.2. Biochemical Characterization

All bacterial samples were Gram-positive, indicating a thick peptidoglycan cell wall without an outer lipid membrane. Biochemical tests showed that all strains were catalase-positive; the catalase enzyme protects cells from oxidative damage by breaking down hydrogen peroxide. All strains tested positive for coagulase, an enzyme that causes blood plasma to clot, indicating virulence potential. The urease test was negative for all strains, showing they could not hydrolyze urea. All strains tested positive for the indole test, indicating that they produce indole by breaking down tryptophan via the enzyme tryptophanase. Finally, the oxidase test was negative for all strains, indicating the absence of cytochrome oxidase enzyme. These chemical characteristics are consistent with standard bacterial identification criteria described in Bergey’s Manual of Systematic Bacteriology and Microbiological Studies.

### 3.3. Bacterial Isolates

Four distinct bacterial colonies were isolated from petroleum-contaminated soil and screened for their ability to treat produced water by growing on mineral salt medium. All four isolates were found to be Gram-positive rods. Biochemical identification suggested the presence of species such as *Bacillus, Vibrio, Spirillum*, and others. All isolates tested positive for the Gram reaction.

### 3.4. Effect of Petroleum Concentration on Bacterial Growth

**Figure 7:**
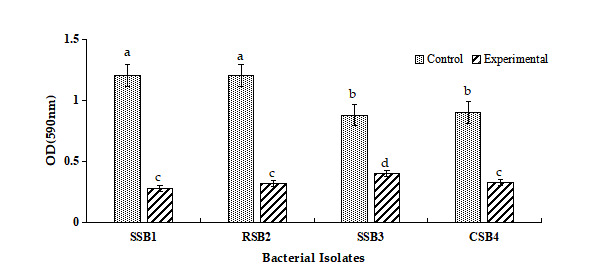
Optical density of bacterial isolates. Different lowercase letters above the bars indicate statistically significant differences among means; bars sharing the same letter are not significantly different at *p* < 0.05.

Produced water negatively affected microbial growth. The effect was assessed by adding 50 µL of produced water to bacterial cultures and measuring optical density (OD) at 590 nm. Four strains—SSB1, RSB2, SSB3, and CSB4—were analyzed. According to the data, the optimal petroleum concentration for growth was below 3%, with SSB1 showing the highest absorbance (1.3 nm). Figure 7 shows the OD values for each isolate.

Out of the four isolates, only two were capable of degrading petroleum hydrocarbons effectively. These bacteria likely possess specific enzymes and genes that enable them to survive and thrive in petroleum-contaminated environments, giving them a competitive advantage over non-degrading strains. This aligns with previous findings [29] that microorganisms are key hydrocarbon degraders, with several *Bacillus* species, such as *Bacillus subtilis* and *Bacillus cereus*, being known for their petroleum degradation capabilities [30,31]. Recent studies have also highlighted the role of *Bacillus* spp. in hydrocarbon bioremediation due to their production of biosurfactants and catabolic enzymes that enhance pollutant breakdown [32,33,34]. The low survival rate of bacteria in petroleum-contaminated soil reflects the toxic effect of hydrocarbons on microbial populations, with only about 0.2% of bacteria persisting under such conditions [35]. This indicates that petroleum contamination significantly limits bacterial growth, likely due to the toxic effects of hydrocarbons. However, certain bacterial strains, particularly *Bacillus* species, exhibited a notable ability to degrade petroleum compounds, suggesting their potential use in bioremediation of petroleum-contaminated soils. The optimal petroleum concentration for growth below 3% implies that high pollutant levels may inhibit microbial activity, highlighting the importance of managing contamination levels to enhance biodegradation efficiency [36].

### 3.5. Microbial-Assisted Phytoremediation

Water hyacinth was cultivated under various petroleum contamination levels to assess the plant’s resilience. Multiple growth parameters, including shoot height, fresh weight, dry weight, root length, and growth rate, were measured over a 15-day period. This study revealed that water hyacinth exhibited a dose-dependent response to petroleum contamination. Although initial growth was noticeably reduced, microbial amendments helped mitigate these adverse effects. These findings align with studies showing that water hyacinth can tolerate moderate levels of petroleum contamination and that microbial interactions can alleviate phytotoxic effects [24,37]. The ability of water hyacinth to sustain growth under such stress conditions supports its potential use in phytoremediation strategies for petroleum-contaminated aquatic environments.

### 3.6. Comparative Analysis of Shoot and Root Length

**Figure 8:**
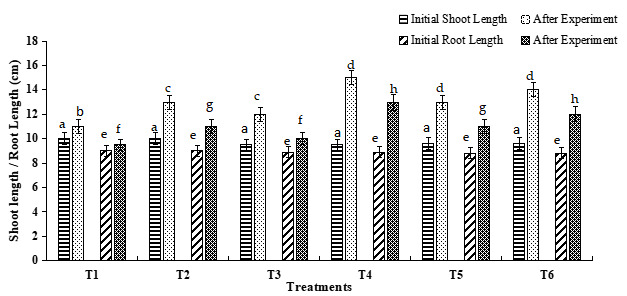
Shoot and root length of water hyacinth before and after treatment. T1 = 100% produced wastewater with *E. crassipes*; T2 = 100% wastewater, plants, and bacterial inoculum; T3 = 75% wastewater with plants only (to evaluate dilution effects and plant-only remediation as a control); T4 = 75% wastewater with plants and bacteria; T5 = 50% wastewater with plants; T6 = 50% wastewater with plant and bacterial inoculum. Different lowercase letters above the bars indicate statistically significant differences among means; bars sharing the same letter are not significantly different at *p* < 0.05.

The combined data for shoot and root development indicated a steady recovery and growth trend across all treatments (T1–T6) (Figure 8). After the 15-day experimental period, both parameters showed a measurable increase compared with their initial values. Shoot length showed significant progress (*p* < 0.05), particularly in treatment T4, reaching an approximately 15 cm, which showed a 12.4% increase compared to initial measurements. This pattern is consistent with the findings of Balasubramaniyam [38], where microbial-assisted recovery helped overcome the growth inhibition typically caused by high hydrocarbon loads [39].

Similarly, root development remained resilient despite petroleum-induced stress. While root growth inhibition often occurs, with the literature highlighting sensitivity to petroleum contaminants due to reduced oxygen [40], the final measurements showed that roots extended up to 13 cm in T4, an increase of up to 18.5% compared with initial measurements. The differential response across treatments emphasizes spatial variability in contamination effects, which is crucial to targeted remediation [41]. Overall, the simultaneous increase in both shoot and root length confirms that the applied microbial treatments successfully countered the toxicity of the environment.

### 3.7. Wet and Dry Weight of Water Hyacinth After Treatment

**Figure 9:**
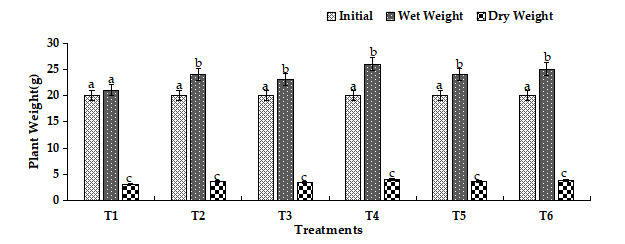
Wet and dry weight of water hyacinth after treatment. T1 = 100% produced wastewater with *E. crassipes*; T2 = 100% wastewater, plants, and bacterial inoculum; T3 = 75% wastewater with plants only (to evaluate dilution effects and plant-only remediation as a control); T4 = 75% wastewater with plants and bacteria; T5 = 50% wastewater with plants; T6 = 50% wastewater with plant and bacterial inoculum. Different lowercase letters above the bars indicate statistically significant differences among means; bars sharing the same letter are not significantly different at *p* < 0.05.

The analysis of the biomass of water hyacinth, measured in terms of wet and dry weights, showed a consistent increase across all treatments (T1–T6) following the 15-day experimental period. Initial plant weight was recorded at approximately 20 g for all groups (Figure 9). By the end of this study, the wet weights ranged from approximately 21 g to 24 g, with treatment T4 exhibiting the highest biomass accumulation, reaching nearly 24 g, which is an increase of about 15.6%. This upward trend in wet weight indicates that the plants successfully recovered from initial petroleum-induced stress and continued to gain mass. The dry weight followed a similar positive trajectory, staying consistently around 3 g to 5 g across the different treatment groups, which corresponds to increases of up to 40%. These biomass trends mirror observations in similar phytoremediation studies where microbial amendments aid in biomass retention and overall plant health, even in contaminated environments [8,42]. The steady maintenance of dry weight suggests that the nutrient uptake and metabolic processes of the water hyacinth were not critically impaired, reinforcing the effectiveness of microbial enhancement in sustaining plant growth under petroleum stress. Two-way ANOVA revealed that treatments had a significant effect on plant weight (*p* < 0.05). The results demonstrate that plant weight is strongly influenced by treatment type, growth stage, and their interaction. In addition, a pronounced interaction effect indicates that treatment efficacy varies across developmental stages, highlighting the importance of considering growth stages when evaluating treatment responses in plants.

### 3.8. Percentage Removal of Heavy Metals in Produced Wastewater

Produced wastewater contained seven heavy metals: Ag, As, Cr, Co, Fe, Ni, and Hg. The percentage removal of these metals varied across treatments, with the highest removal being observed in T1 and the lowest in T4 and T6 (Table 2). Mercury showed the lowest removal efficiency (18.5–33.3%) across all treatments, highlighting its persistence and toxicity. The other metals, i.e., Ag, As, Cr, Co, Fe, and Ni, showed higher removal rates, with Ag reaching up to 79.2% in T1 and Ni up to 64%. The decreasing trend in removal efficiency from T1 to T6 suggests that higher contamination levels inhibit the phytoremediation process. Notably, T3 recorded the highest Co removal (79%), indicating that the combined treatment of plant, microbes, and amendment was effective for certain metals. These results confirm that water hyacinth, supported by microbial activity, is effective in removing heavy metals from contaminated water. However, metals like Hg and Fe are more resistant, possibly due to low bioavailability or chemical stability. The decline in removal efficiency under severe contamination (T6) highlights the need for controlled pollutant levels and tailored microbial–plant systems for optimal performance [9,43,44]. The removal efficiency differed significantly among the six water treatment methods. This indicates that the choice of treatment method has a strong influence on heavy metal removal percentage (*p* < 0.05). The removal percentage also differed significantly among the seven heavy metals. Some metals were removed more efficiently than others, regardless of the treatment method (*p* < 0.05). The effectiveness of a given water treatment method depends on the type of heavy metal [5]. In other words, there is a strong interaction between treatment method and metal type. Overall, this suggests that microbial-assisted phytoremediation offers a promising though metal-specific strategy for treating produced wastewater.

### 3.9. Limitations of This Study

This study did not include plant-free or sterile-inoculum controls nor molecular microbial identification (e.g., 16S rRNA sequencing). Therefore, microbial contributions cannot be isolated, and related interpretations are presented cautiously.

It should be noted that part of the observed reductions in metal concentrations and EC/TDSs may result from dilution, sedimentation, and physicochemical processes rather than exclusively from biological activity.

Future studies should incorporate hydrocarbon quantification (TPH/GC–MS), comprehensive mass-balance analyses, rigorous control treatments, and long-term validation to distinguish biological versus physicochemical contributions while assessing remediation sustainability and mechanisms.

## 4. Conclusions

Phytoremediation has been found to be an effective and sustainable method for treating contaminated water, especially when microbial assistance is provided. In this study, contaminant-tolerant bacterial isolates were successfully obtained from produced water contaminated soil from an oil field site. Eichhornia crassipes (water hyacinth) was effective in reducing key physicochemical parameters, including pH, TDS, EC, bicarbonates, and chloride levels, and demonstrated significant removal of heavy metals in produced water within 15 days. The overall pollutant removal, focusing on heavy metals and salinity, was substantial. The plant was also efficient in post-biofilter wastewater treatment. Although promising removal performance was observed, the absence of sterile controls and molecular microbial analysis limits definitive attribution of microbial effects. These results suggest that water hyacinth, particularly when combined with microbial augmentation, could be a valuable tool in future environmental cleanup efforts focused on heavy metal and salinity reduction in produced water.

## Figures and Tables

**Table 1:** Analysis of biochemical tests for species characterization of isolated microbial strains.

No. of Strains	Shapes	Gram Staining	Catalase Test	Urease Test	Indole Test	Coagulase Test	Oxidase Test
1	Spiral-shaped	+	+	−	+	+	−
2	Rod-shaped	+	+	−	+	+	−
3	Spherical	+	+	−	+	+	−
4	Comma-shaped	+	+	−	+	+	−

**Table 2:** Average percentage removal of heavy metals in produced wastewater.

Samples	Ag %	As %	Co %	Cr%	Fe%	Hg%	Ni%
T1	79.2 ± 0.02	70.1 ± 0.01	60 ± 0.01	50 ± 0.01	34.3 ± 0.01	33.3 ± 0.03	64 ± 0.01
T2	62.2 ± 0.01	60 ± 0.01	60 ± 0.01	49 ± 0.01	53.1 ± 0.01	31.1 ± 0.02	60 ± 0.01
T3	67 ± 0.01	35 ± 0.01	79 ± 0.01	50 ± 0.01	46.8 ± 0.01	40 ± 0.02	54 ± 0.01
T4	11.1 ± 0.01	31.2 ± 0.01	49 ± 0.01	42 ± 0.01	28.1 ± 0.01	18.5 ± 0.02	32 ± 0.02
T5	66.6 ± 0.01	45 ± 0.01	49 ± 0.01	44 ± 0.01	59 ± 0.01	22 ± 0.03	37 ± 0.02
T6	60 ± 0.01	66.6 ± 0.01	60 ± 0.01	43.5 ± 0.01	58 ± 0.01	23 ± 0.02	54 ± 0.02

T1 = 100% produced wastewater with *E. crassipes*; T2 = 100% wastewater, plants, and bacterial inoculum; T3 = 75% wastewater with plants only (to evaluate dilution effects and plant-only remediation as a control); T4 = 75% wastewater with plants and bacteria; T5 = 50% wastewater with plants; T6 = 50% wastewater with plant and bacterial inoculum.

## Data Availability

The raw data supporting the conclusions of this article will be made available by the authors upon request.
